# Supplementary material for: A robust method for measuring aminoacylation through tRNA-Seq
Source: eLife. 2024 Jul 30;12:RP91554. doi: 10.7554/eLife.91554 (PMC11288633; doi:10.7554/eLife.91554)
Supplement: Figure 2—source data 2. [file elife-91554-fig2-data2.zip › Original files for images in figure 2/A_LE.pdf]

oxidation_test_8_long-exp

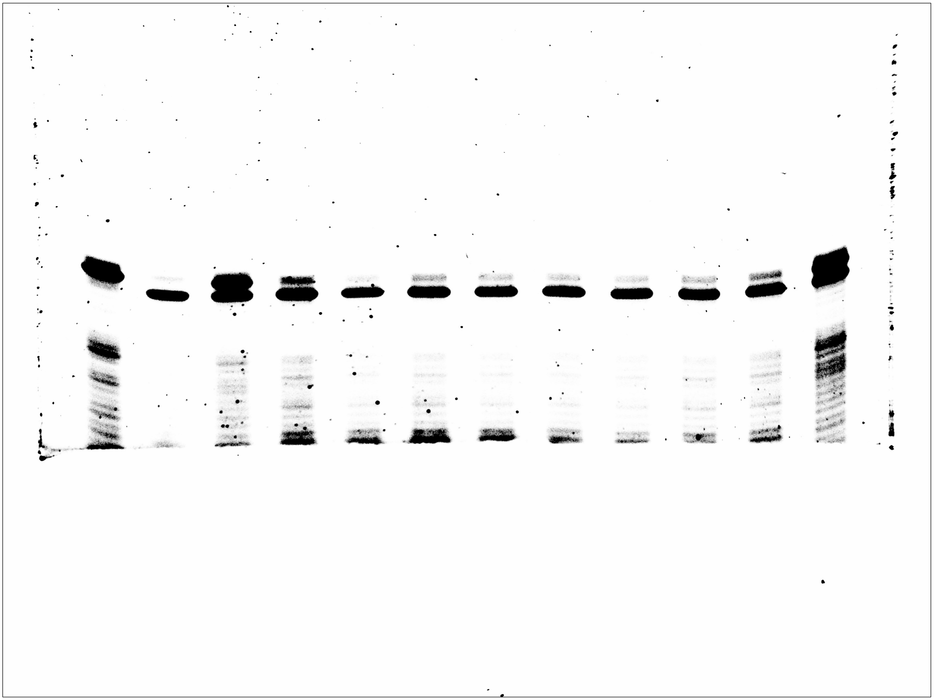

C:/Users/mshared/Desktop/Sullivan Lab/krdav/tRNAseq_opti/oxidation_test
