# Supplementary figures and images for: A robust method for measuring aminoacylation through tRNA-Seq
Source: eLife. 2024 Jul 30;12:RP91554. doi: 10.7554/eLife.91554 (PMC11288633; doi:10.7554/eLife.91554)

## oxidation\_test\_8

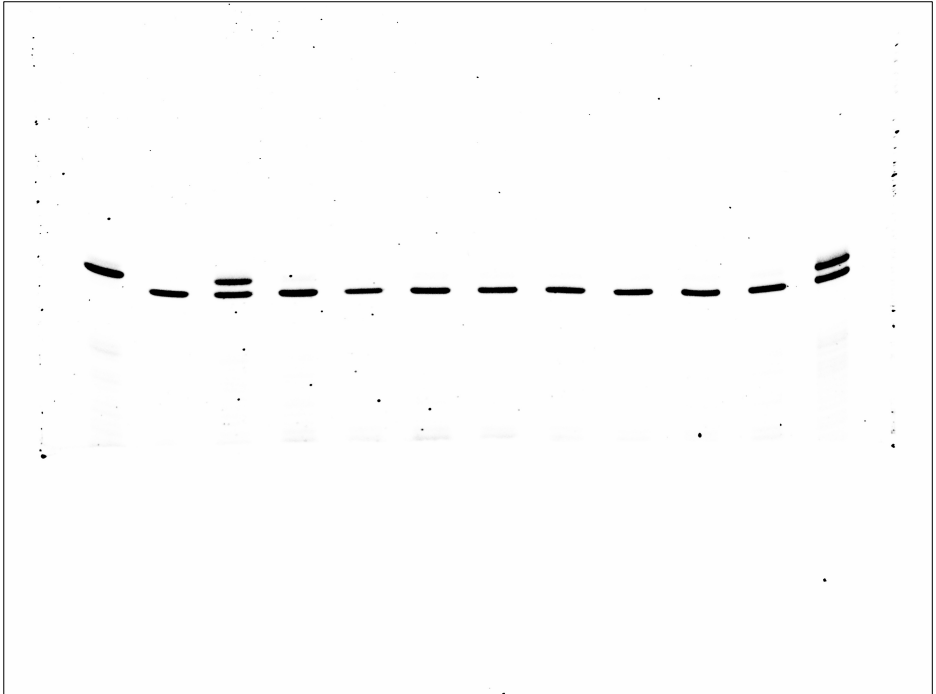

Supplement: Figure 2—source data 2. [file elife-91554-fig2-data2.zip › Original files for images in figure 2/A_SE.pdf]

# Lysine-cleavage\_time\_07-22-23

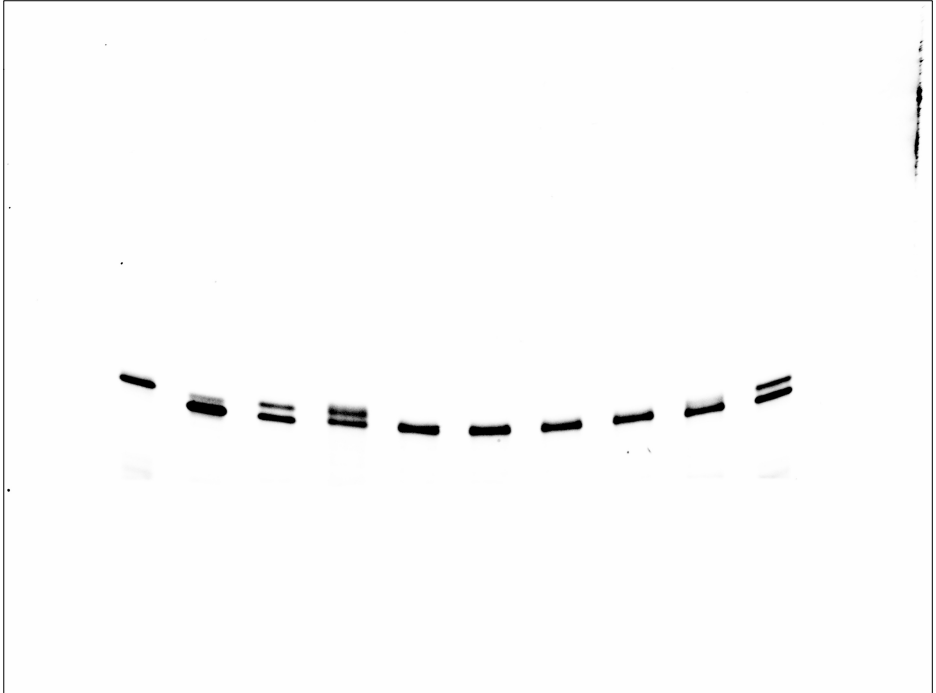

Supplement: Figure 2—source data 2. [file elife-91554-fig2-data2.zip › Original files for images in figure 2/B.pdf]

## ligation-test\_l1Sp

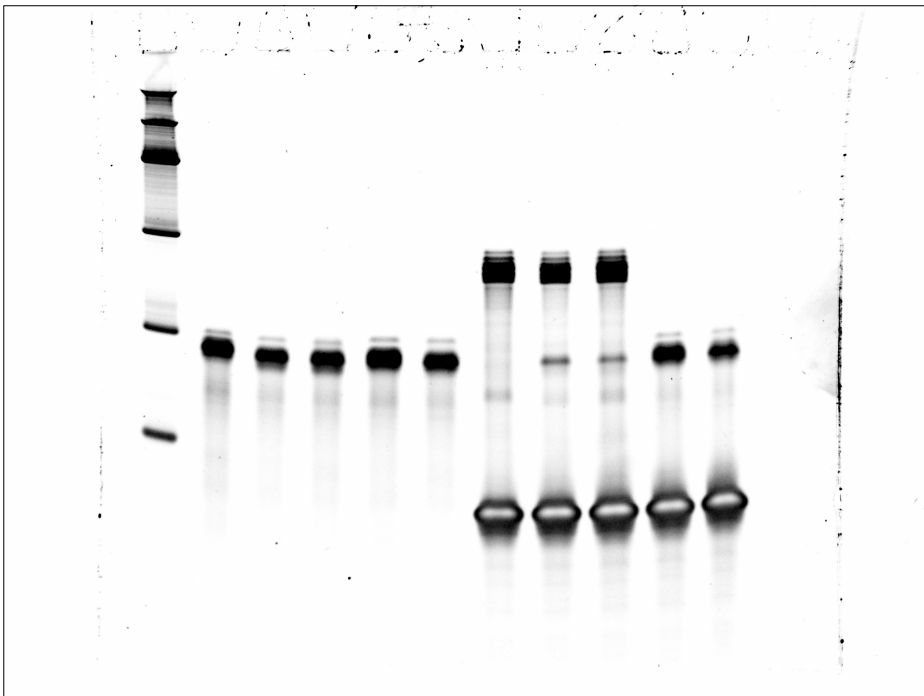

Supplement: Figure 2—source data 2. [file elife-91554-fig2-data2.zip › Original files for images in figure 2/E.pdf]

# ecoli-Lys\_cleavage-test

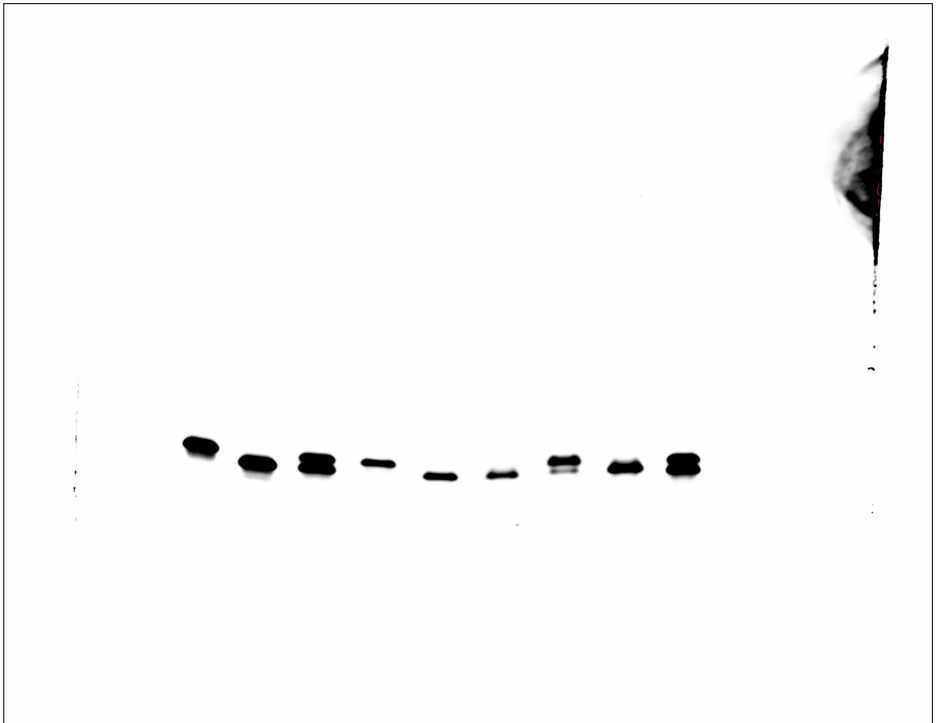

Supplement: Figure 2—source data 2. [file elife-91554-fig2-data2.zip › Original files for images in figure 2/D.pdf]

pH-opti

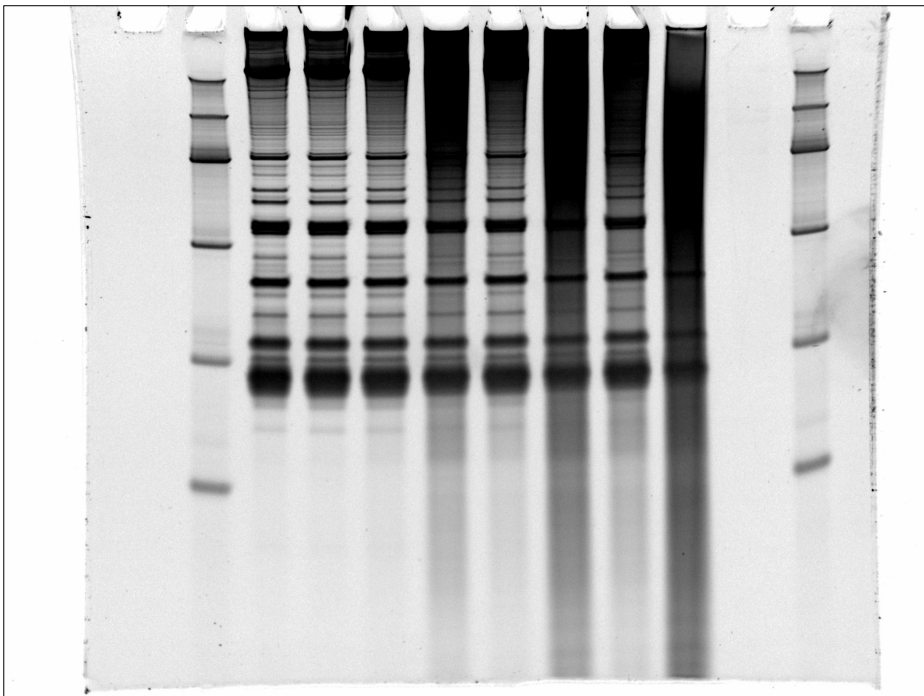

Location: C:/Users/mshared/Desktop/Sullivan Lab/krdav/tRNAseq\_pub/pH

Printed: 3/30/2023 6:11 PM

Page 1 of 1

Supplement: Figure 2—figure supplement 1—source data 3. [file elife-91554-fig2-figsupp1-data3.zip › Original files for images in figure 2ΓÇöfigure supplement 1/B.pdf]

## non-complementary-splint-ligation

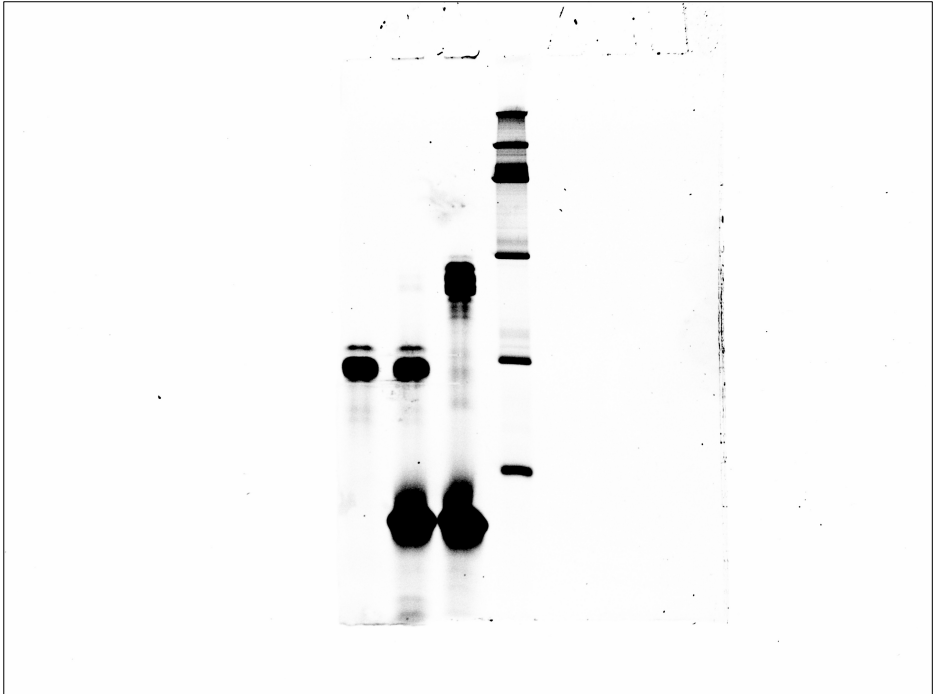

Supplement: Figure 2—figure supplement 4—source data 1. [file elife-91554-fig2-figsupp4-data1.zip › Original files for images in figure 2ΓÇöfigure supplement 4/C.pdf]

## ligation-test\_I3Sp

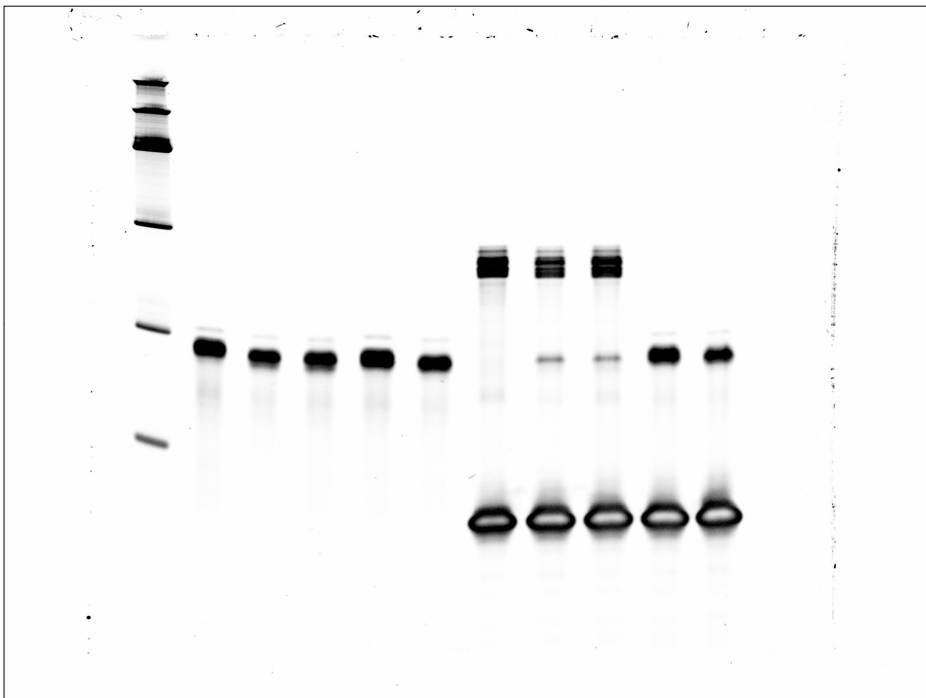

Supplement: Figure 2—figure supplement 5—source data 1. [file elife-91554-fig2-figsupp5-data1.zip › Original files for images in figure 2ΓÇöfigure supplement 5/A_bottom.pdf]

## ligation-test\_I2Sp

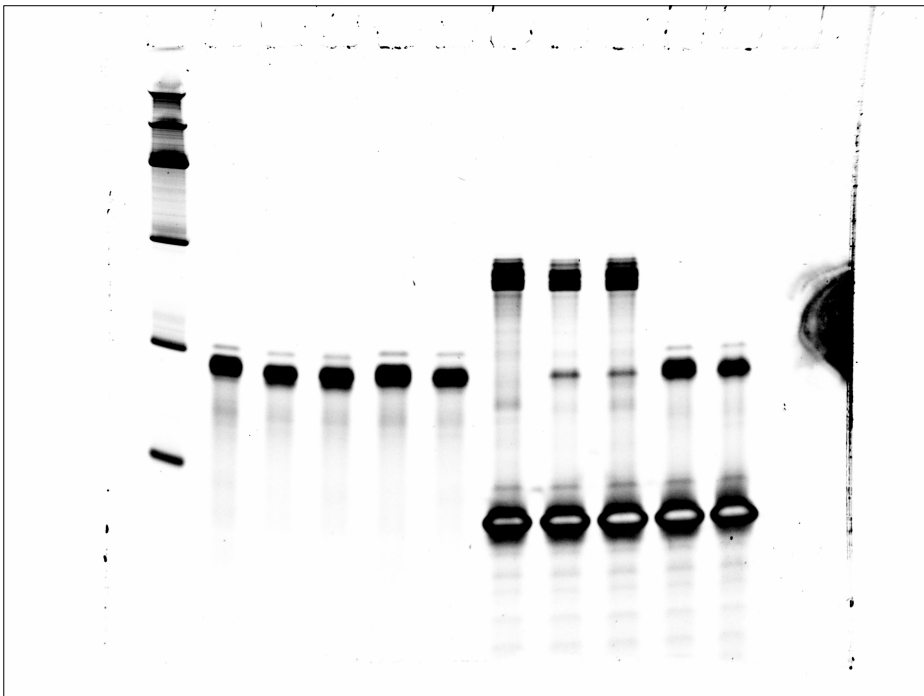

Supplement: Figure 2—figure supplement 5—source data 1. [file elife-91554-fig2-figsupp5-data1.zip › Original files for images in figure 2ΓÇöfigure supplement 5/A_top.pdf]

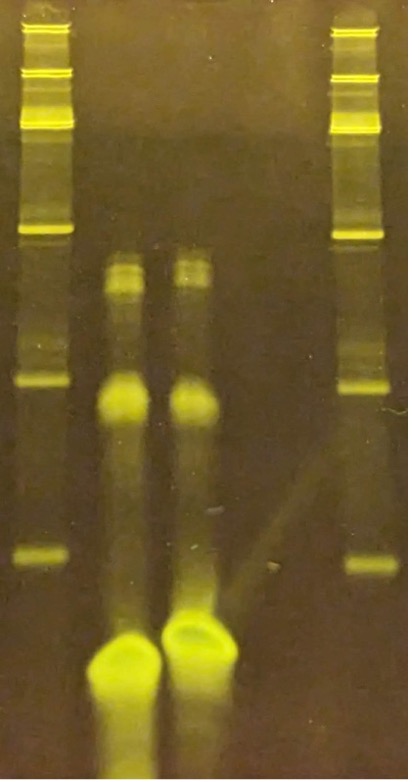

Supplement: Figure 2—figure supplement 5—source data 1. [file elife-91554-fig2-figsupp5-data1.zip › Original files for images in figure 2ΓÇöfigure supplement 5/B.jpg]

## RT-PCR\_test

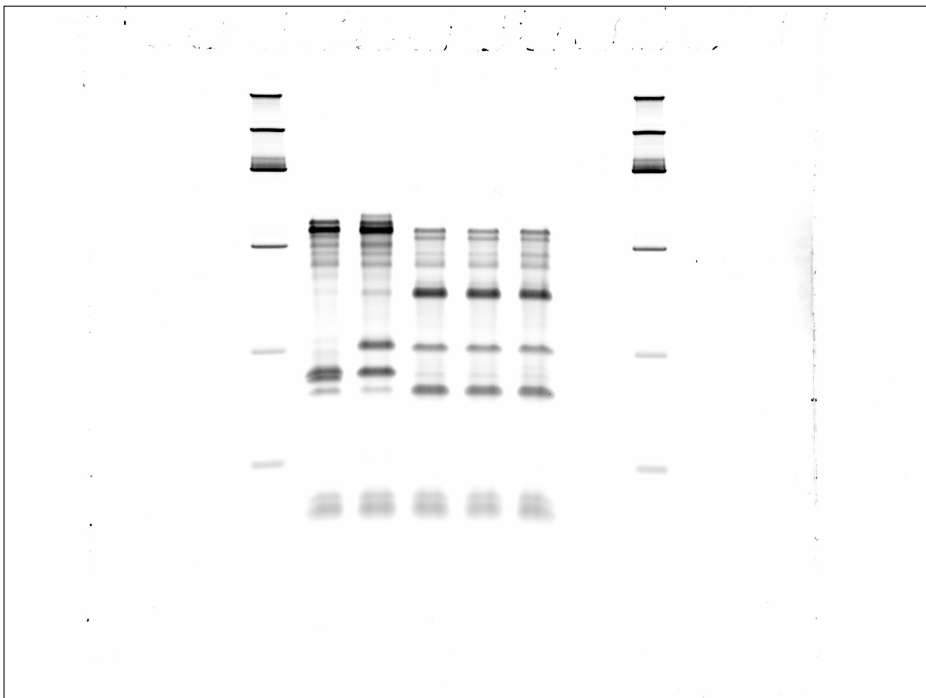

Supplement: Figure 2—figure supplement 6—source data 2. [file elife-91554-fig2-figsupp6-data2.zip › Original files for images in figure 2ΓÇöfigure supplement 6/A.pdf]
