# Supplementary material for: A robust method for measuring aminoacylation through tRNA-Seq
Source: eLife. 2024 Jul 30;12:RP91554. doi: 10.7554/eLife.91554 (PMC11288633; doi:10.7554/eLife.91554)
Supplement: Figure 2—source data 3. [file elife-91554-fig2-data3.docx]

**Figure 2, panel A, short exposure (SE)**

Cropped area marked by red box.

30

10

5

1

Mix

CC

CCA

**Figure 2, panel A, long exposure (LE)**

Cropped area marked by red box.

30

10

5

1

Mix

CC

CCA

**Figure 2, panel B**

Cropped area marked by red box.

30

90

10

0

CCA

CC

Mix

**Figure 2, panel D**

Cropped area marked by red box.

Mix

CCA

CC

**Figure 2, panel E**

Cropped area marked by red box.
