# Supplementary material for: A robust method for measuring aminoacylation through tRNA-Seq
Source: eLife. 2024 Jul 30;12:RP91554. doi: 10.7554/eLife.91554 (PMC11288633; doi:10.7554/eLife.91554)
Supplement: Figure 2—figure supplement 3—source data 1. [file elife-91554-fig2-figsupp3-data1.zip › Original files for images in figure 2ΓÇöfigure supplement 3/C.pdf]

ligation_opti_3

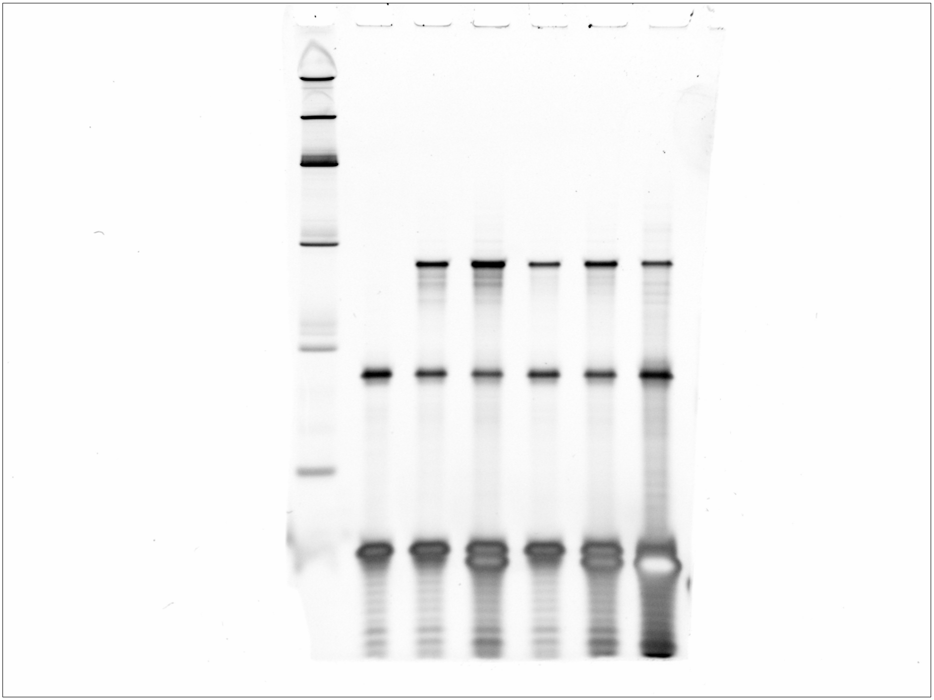

Location: C:/Users/mshared/Desktop/Sullivan Lab/krdav/tRNAseq_opti
Printed: 12/13/2022 6:28 PM
