# Supplementary material for: A robust method for measuring aminoacylation through tRNA-Seq
Source: eLife. 2024 Jul 30;12:RP91554. doi: 10.7554/eLife.91554 (PMC11288633; doi:10.7554/eLife.91554)
Supplement: Figure 2—figure supplement 4—source data 2. [file elife-91554-fig2-figsupp4-data2.docx]

**Figure 2—figure supplement 4, panel A**

Cropped area marked by red box.

**Figure 2—figure supplement 4, panel B**

Cropped area marked by red box.

**Figure 2—figure supplement 4, panel C**

Cropped area marked by red box. After cropping the image was horizontally flipped to place the ladder on the right side.

Input

NCMPL

l1Sp
