# Supplementary material for: A robust method for measuring aminoacylation through tRNA-Seq
Source: eLife. 2024 Jul 30;12:RP91554. doi: 10.7554/eLife.91554 (PMC11288633; doi:10.7554/eLife.91554)

**Figure 2—figure supplement 5, panel A, top**

Cropped area marked by red box.

**Figure 2—figure supplement 5, panel A, bottom**

Cropped area marked by red box.

**Figure 2—figure supplement 5, panel B**

Cropped area marked by red box. After cropping, the image was color inverted to black/white.


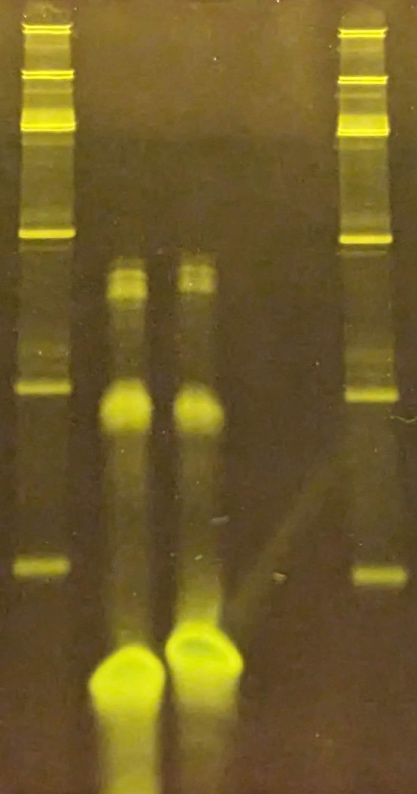

Supplement: Figure 2—figure supplement 5—source data 2. [file elife-91554-fig2-figsupp5-data2.docx]
