# Supplementary material for: A robust method for measuring aminoacylation through tRNA-Seq
Source: eLife. 2024 Jul 30;12:RP91554. doi: 10.7554/eLife.91554 (PMC11288633; doi:10.7554/eLife.91554)
Supplement: Figure 2—figure supplement 6—source data 3. [file elife-91554-fig2-figsupp6-data3.docx]

**Figure 2—figure supplement 6, panel A**

Cropped area marked by red box.
