# Supplementary material for: A robust method for measuring aminoacylation through tRNA-Seq
Source: eLife. 2024 Jul 30;12:RP91554. doi: 10.7554/eLife.91554 (PMC11288633; doi:10.7554/eLife.91554)
Supplement: Figure 5—figure supplement 3—source data 2. [file elife-91554-fig5-figsupp3-data2.zip › Original files for images in figure 5ΓÇöfigure supplement 5/A.pdf]

Thr-CCA-P_PureExpress_ligation-control

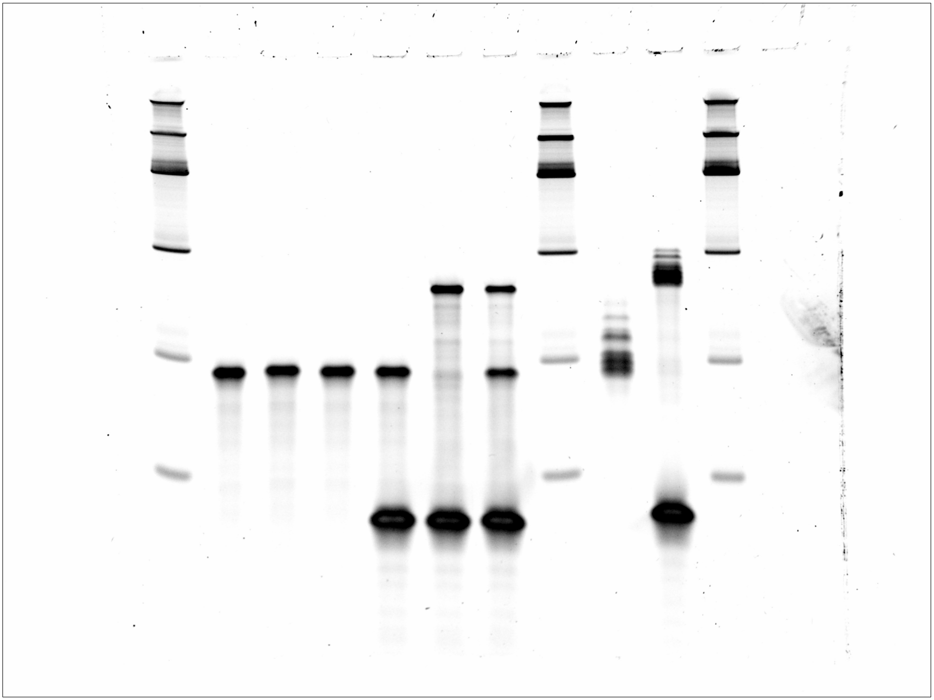

Location: C:/Users/mshared/Desktop/Sullivan Lab/krdav
Printed: 4/11/2023 4:03 PM
