# Supplementary material for: A robust method for measuring aminoacylation through tRNA-Seq
Source: eLife. 2024 Jul 30;12:RP91554. doi: 10.7554/eLife.91554 (PMC11288633; doi:10.7554/eLife.91554)
Supplement: Figure 6—figure supplement 1—source data 2. [file elife-91554-fig6-figsupp1-data2.zip › Original files for images in figure 6ΓÇöfigure supplement 1/A.pdf]

RNA_stability_after_incubation_short-exp

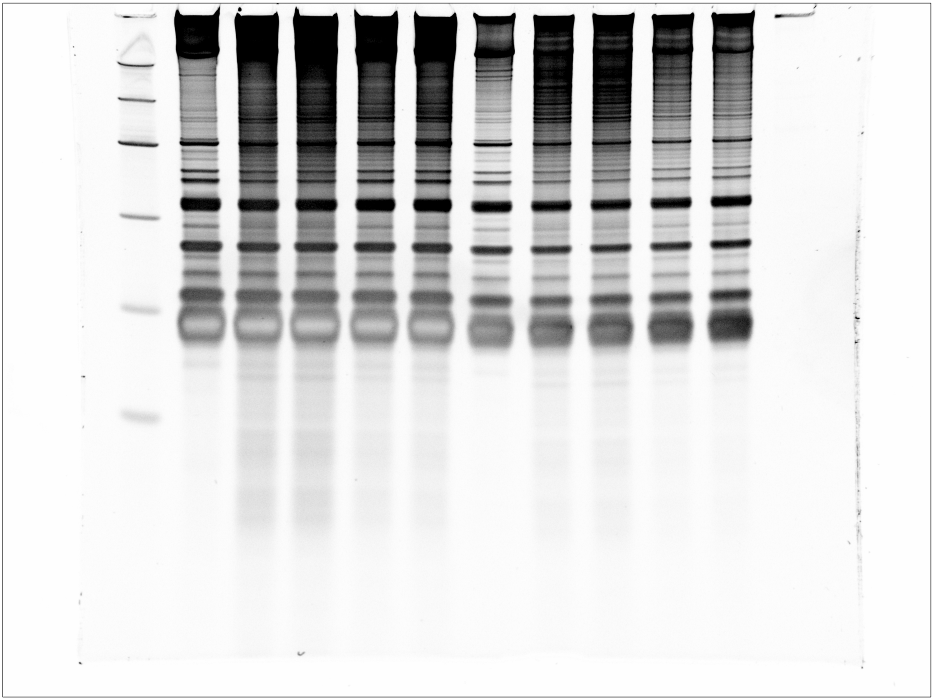

C:/Users/mshared/Desktop/Sullivan Lab/krdav/tRNAseq_tRNA_half-life
Printed: 1/30/2023 4:32 PM
